# Feasibility of Imaging Myelin Lesions in Multiple Sclerosis

**DOI:** 10.1155/2011/953806

**Published:** 2011-08-15

**Authors:** Maria I. Zavodszky, John F. Graf, Cristina A. Tan Hehir

**Affiliations:** General Electric Global Research Center, 1 Research Circle, Niskayuna, NY 12309, USA

## Abstract

The goal of this study was to provide a feasibility assessment for PET imaging of multiple sclerosis (MS) lesions based on their decreased myelin content relative to the surrounding normal-appearing brain tissue. The imaging agent evaluated for this purpose is a molecule that binds strongly and specifically to myelin basic protein. Physiology-based pharmacokinetic modeling combined with PET image simulation applied to a brain model was used to examine whether such an agent would allow the differentiation of artificial lesions 4–10 mm in diameter from the surrounding normal-looking white and gray matter. Furthermore, we examined how changes in agent properties, model parameters, and experimental conditions can influence imageability, identifying a set of conditions under which imaging of MS lesions might be feasible. Based on our results, we concluded that PET imaging has the potential to become a useful complementary method to MRI for MS diagnosis and therapy monitoring.

## 1. Introduction

Multiple sclerosis (MS) is a progressively debilitating neurological disease first described in the mid-19th century [1]. It is named after the multiple lesions of demyelinated white matter in the central nervous system (CNS) of MS patients. Between 1980 and 2000, MS was diagnosed based on clinical symptoms, paraclinical evidence (MRI, urodynamics, electroencephalography potentials measured after visual stimuli, also called visual evoked potentials), and immunoglobulin abnormalities of the cerebrospinal fluid (CSF), according to the criteria developed by the National Multiple Sclerosis Society [2]. In 2000, another committee of the Society updated the MS diagnostic criteria. Modern diagnosis of MS requires additional evidence of lesions in the CNS, disseminated in time and space, provided primarily by MRI.

MS is the most common demyelinating disease, affecting approximately 350,000 persons in the USA alone. The average age at the onset of MS is 32 years, and patients usually live 35 years after the diagnosis [3]. The total cost of MS is estimated at $47,215 per patient and year according to a study from 2005 (http://ideas.repec.org/p/hhs/hastef/0594.html). Because disease-modifying drugs account for about 34% of the total cost, developing sensitive and specific methods for monitoring the effectiveness of such treatments would be highly desirable. This, however, has proven to be difficult. The reason is the need to extrapolate the results of relatively short-term studies to the long-term course of MS which is naturally quite variable with acute attacks followed by relative stability and/or steady progression [3]. Instead of evaluating changes in clinical symptoms, more recent clinical trials have been aimed at obtaining physical evidence generated by MRI for positive changes as a result of the treatments. 

Myelin basic protein (MBP), the proposed target of the small molecule agent evaluated for PET imaging in this study, is the second most abundant protein in the central nervous system (CNS) myelin after proteolipid protein (PLP). It accounts for approximately 25–30% of the total protein content and 10% of the dry weight of myelin [4]. It is an extrinsic membrane protein attached to the cytoplasmic side of the oligodendrocyte membrane. Given their role of myelin-producing cells, oligodendrocytes are mostly found in the white matter, but they also occur in the gray matter, providing the myelin sheets for axons traversing the gray matter. As a consequence, MBP can also be found in the gray matter, although its concentrations in the white matter is 7-8 times higher [5]. 

MS is considered to be an inflammatory disease in which inflammation of the blood vessels in the CNS leads to the destruction of myelin sheets covering the axons of the neurons. The hallmark of MS is the presence of lesions in which myelin sheaths are damaged to varying degrees. In the same areas, axons are still present and appear undamaged, embedded in a dense astroglial tissue that also contains lymphocytes and macrophages. MS lesions are very heterogeneous with regards to size, composition, location, and possibly even mechanism of formation. They can be found anywhere in the CNS but have been most frequently detected in the optic nerve, in the deep cerebral white matter (especially around the ventricles), in the cerebellar peduncles, and certain parts of the brainstem and spinal cord. Old chronic plaques are gray, hard and sharply demarcated. Fresh lesions, still in process of myelin destruction, are yellow to brown and of soft consistency. It is important to note that the lesions do not completely lack myelin. Myelin loss in these lesions can range from 10–90%. In very severe cases, there could be more severe tissue destruction leading to cystic loss of tissue, mainly in the center of the lesions [3]. From the point of view of the present study, varying myelin content has to be taken into account when estimating the target concentration in the lesions. 

Even though the plaques have a relatively high concentration of sclerotic tissue, they maintain a significant vascular component. Furthermore, increased number and size of blood vessels were reported in acute lesions. It has been hypothesized that angiogenesis plays a significant role in promoting the disease progression by delivering the agents maintaining the inflammation around blood vessels and venules [7]. This also means that maintained blood flow will allow delivery of the imaging agent to the lesions.

**Figure 1:**
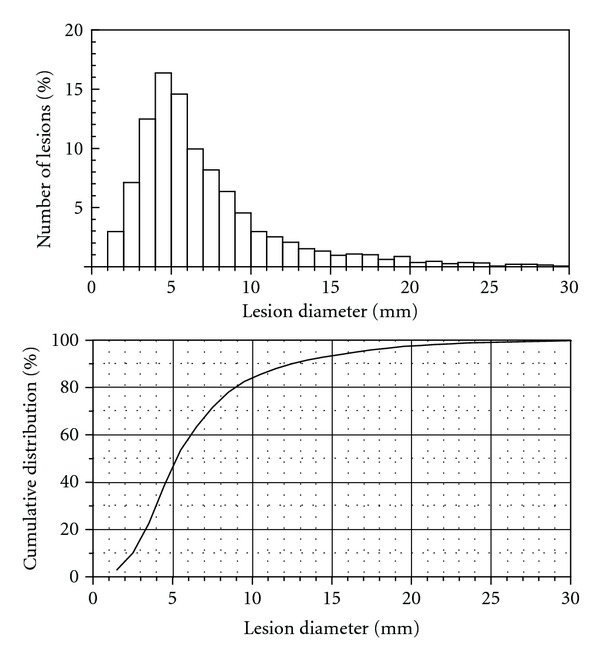
The distribution of lesions according to their sizes in 28 MS patients. The plot has been reproduced based on the findings reported by Wang and colleagues and contains data on both SPMS and RRMS forms of MS [6].

In an effort to analyze the size distribution of MS lesions, Wang and coworkers surveyed the T2 weighted brain MRI images of 28 patients, 15 with secondary progressive form (SPMS) and 13 with relapsing remitting form of MS (RRMS) [6]. Myelin lesions were identified by experienced observers using a contouring technique. SPMS patients were found to have more but smaller lesions compared to RRMS patients. Overall, 60% of the examined 2766 lesions had diameters between 3.5 and 9 mm, 20% were larger than 9 mm and 20% smaller than 3.5 mm (Figure 1). Based on these findings, lesion sizes were modeled to be 4–10 mm in diameter in this study. 

**Figure 2:**
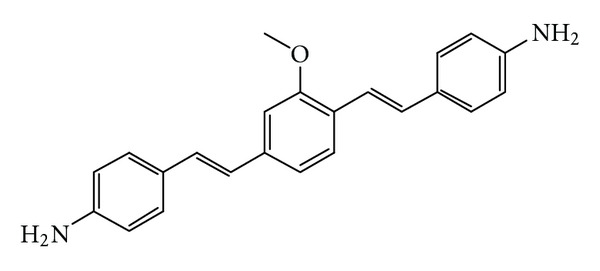
Imaging agent candidate BMB.

Currently, MRI is the standard method for imaging MS lesions. Conventional MRI metrics are routinely used to improve the diagnosis of MS and to monitor the effects of therapy. T2-weighted MRI imaging is very effective in identifying MS lesions. However, the areas of increased signal seen on T2-weighted MRI images reflect increased water content, which is not specific for MS and cannot provide information about the degree of myelin destruction in the lesions. This is because other pathological processes resulting in inflammation or tissue loss in the brain can result in increased signal [8, 9]. Another technique, T1-weighted imaging following the administration of gadolinium-DTPA, has been reported to increase the lesion detection rate, but its use depends on the presence of a leaky blood-brain barrier [10–12]. Gadolinium-enhanced MRI studies of patients with early RRMS were able to detect disruption of the blood-brain barrier indicating that this method provides a sensitive measure of at least one aspect of the disease [13, 14]. However, only weak correlation was found between MRI metrics (new/enlarging T2 lesions and gadolinium enhancing lesions, T2 lesion load, T1 hypointense lesions) and clinical subtypes and symptoms [15]. It is highly desirable to have an additional method, such as PET imaging, with high specificity for MS lesions. Combined with the sensitivity of MRI, it has the potential to become a practical therapeutic tool.

(E,E)-1,4-bis(p-aminostyryl)-2-methoxy-benzene (BMB) is a small molecule that was reported to bind specifically and with high affinity to MBP (Figure 2) [16, 17]. Stankoff and coworkers showed that in vitro labeling of postmortem brain sections allows the identification of MS lesions [16]. They also demonstrated that BMB penetrates the blood-brain barrier by imaging CNS myelin of baboons by PET using ^11^C-labeled BMB as a marker. The study showed a higher retention rate of BMB in the subcortical matter than in the adjacent cortex. However, the difference was only about 10–20%. The authors speculate that this might be due to the presence of some myelinated fibers and the blood-flow-dependent delivery of BMB to the highly vascularized areas of the gray structures [16]. Due to its specificity for myelin and its ability to cross the blood-brain barrier, BMB or a related molecule with similar properties (labeled with an appropriate tracer) might be usable as an agent for imaging MS lesions with PET. This approach would complement MRI imaging by providing the desired specificity to differentiate between lesions of various pathological origin detectable by MRI and to monitor treatment aimed at promoting remyelination. 

**Figure 3:**
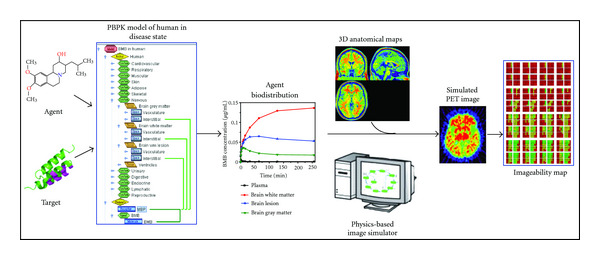
Schematic workflow of an imaging feasibility study.

The purpose of this work was to assess whether BMB or a similar agent binding specifically to a component of the myelin would allow the detection of MS lesions with PET. We used a detailed mechanistic physiology-based pharmacokinetic (PBPK) modeling tool to simulate the biodistribution of the agent in every tissue as a function of time in a human model with 4–10 mm demyelinated lesions in the white matter of the brain. The agent pharmacokinetics is crucial for imaging since the overall balance of competing effects such as specific binding to the target, nonspecific binding to surrounding tissues, delivery, and clearance—all within a narrow timeframe—will determine image quality. The predicted time-dependent agent concentration data and a detailed anatomical phantom were used as the input to a PET image simulator to generate images of the model brain with the MS-type lesions. The images were analyzed to establish imageability criteria that were then used to define agent and physiology property ranges required for successful imaging (Figure 3).

## 2. Methods

### 2.1. PBPK Software

The distribution of BMB in the human body was simulated with the PBPK modeling software BioDMET [18], developed at GE Global Research to aid the development of imaging agents. Ordinary differential equations represent the circulation of body fluids through organs and tissues (macroscopic scale) and the biological transport mechanisms and biotransformations within cells and their organelles (molecular scale). Each major organ in the body is modeled as composed of one or more tissues. The tissues of the model are made up of cells and fluid spaces. The model accounts for the circulation of arterial and venous blood as well as lymph. The use of a PBPK computational model enables the inclusion of kinetic effects that are critical in proper assessment of molecular imaging feasibility. Examples of such kinetic effects include agent delivery to the target location, the competition between target and background binding rates, partitioning of the agent in the various organs, and compartments within organs, as well as biliary and renal clearance rates. 

The BioDMET software's ability to predict drug concentrations has been tested using published data on 26 pharmaceuticals in 45 individual human and animal models. Good correlation was obtained between experimentally measured and calculated log concentrations of drugs/agents in plasma (*R*^2^ = 0.93) and in various other tissues (*R*^2^ = 0.89). The standard deviation in the Log_10 _(measured/calculated) ratios was 0.39 with a mean value of 0.08 for the plasma, and 0.45 with a mean value of 0.13 for the tissues (Graf et al., manuscript in preparation). This level of predictive accuracy is similar to that found with other PBPK models [19].

### 2.2. PBPK Model Input Parameters

The whole body physiology model of the human organism provided with BioDMET was customized to include the relevant anatomical details for MS. The brain was modeled as having three compartments: white matter, gray matter, and lesions. The water, protein, and lipid composition of these compartments was set to correspond to the values found in the literature [20–22]. Concentration values of MBP in the three compartments used in this study are averages of the MBP concentrations measured in various brain regions by radioimmunoassay [23, 24]. The molecular weight of various MBP isoforms varies between 17.2–21 kDa. The molecular weight of the major human isoform, 18.5 kDa, was used in this study [25]. These and other input parameter values are listed in Tables 1 and 2. 

### 2.3. Phantom with MS Lesions

**Figure 4:**
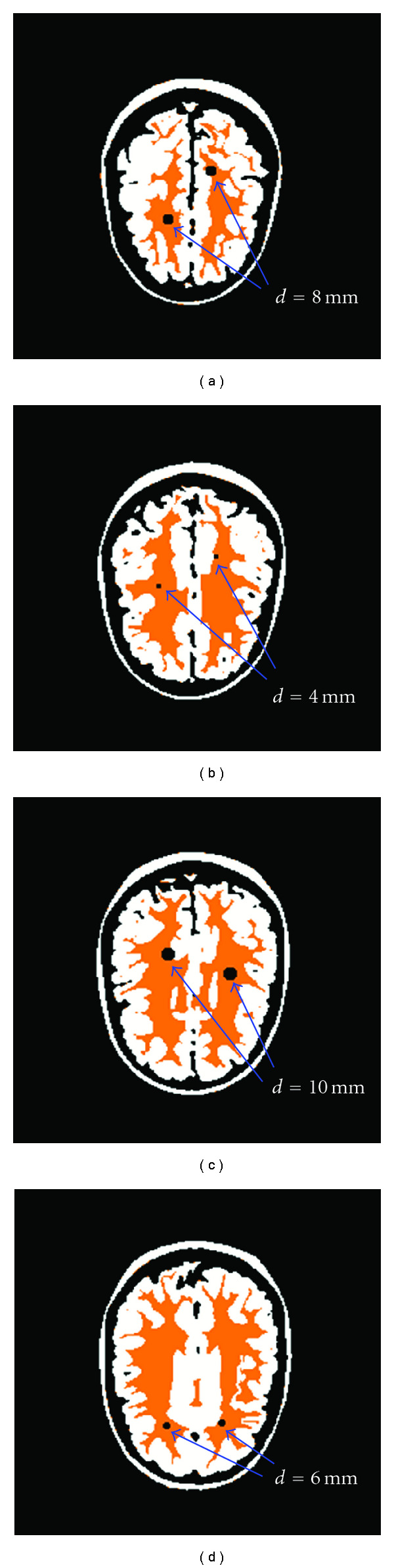
Blue arrows point at lesions inserted into the Zubal head phantom at different transverse locations: (a) slice 45, (b) slice 52, (c) slice 58, and (d) slice 63. According to the coloring scheme of MRIcro, white matter is shown in orange, gray matter in white, and lesions in black.

Artificial lesions of representative sizes for MS lesions (spheres of diameters of 4, 6, 8, and 10 mm) were inserted into the white matter of the Zubal head phantom [26] (Table 3). To assess the imageability of lesions as a function of location, every lesion of a certain size was inserted both into a region completely surrounded by white matter and into another region situated at the border of white and gray matter in the same slice (Figure 4). To locate appropriate regions and identify their coordinates, the head phantom was visualized with the program MRIcro [27].

### 2.4. Image Simulator

Using the time-activity curves calculated with BioDMET and the parameters of the head phantom, PET images were simulated for realistic ranges of conditions (Table 4) with a program developed at GE GRC to aid in the design and analysis of system geometries and image generation chains. The PET image simulator used for the present work is an analytical simulation framework designed for system-level and image reconstruction algorithm comparisons [28]. It is based on accurate physical and statistical models of imaging systems and can generate system models that contain the detection probabilities for photons originating at different locations within the subject. These probabilities are precomputed and stored for use in data generation. Physical events that corrupt the data, such as scattering and random (PET only) events, as well as attenuation within the patient and variable detector efficiencies are also taken into account for more accurate simulations of real imaging systems. Statistical noise that is present in the data, and is often a limiting factor for the resolution/noise tradeoffs in the imaging systems, is modeled as additive, independent Poisson noise. The final system model is then calibrated to directly relate activity concentrations within the subject to counts measured at each detector element. The following input acquisition parameters were used for the PET simulator:

- interval between time points of the time-activity measurement: 2 min,
- scan duration: 20 min,
- injected dose: 10 mCi,
- time between injection of imaging agent and scan start: 2 h,
- half life of radioisotope: 6588 s,
- % Positron yield of radio-isotope: 0.97.

### 2.5. Image Analysis to Determine Signal-to-Noise Ratios

**Figure 5:**
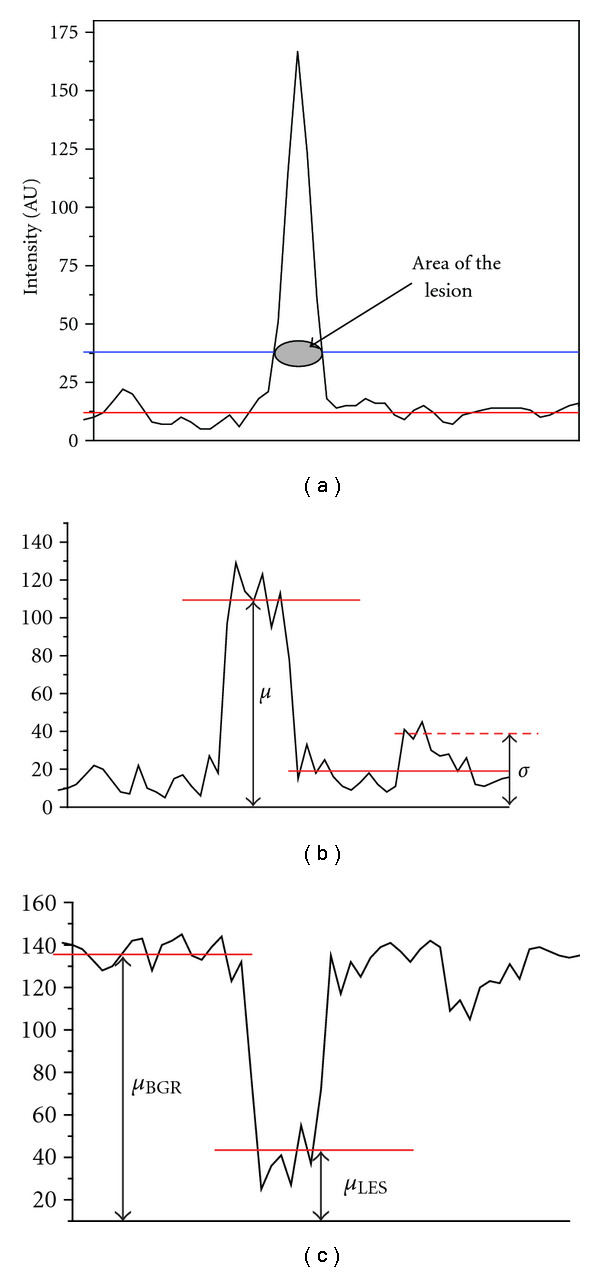
Defining the area of a lesion (a). Positive (b) and negative (c) signals.

Slices of simulated PET images dissecting the regions at the center of the lesions were analyzed with the program OriginPro 8. To assess imageability, the signal-to-noise ratio was calculated for each lesion the following way. Images with activities modeled only in the lesions were used to define the location and area of the lesions on the simulated images. Pixels were counted as part of the lesion if the signal intensity was above the cutoff defined by:

(1) $$\begin{array}{c} \text{Cutoff}=\frac{\;\text{MaxSignal}-\text{Baseline}}{4}, \end{array}$$

where the baseline is the mean signal intensity calculated over the entire image. This is equivalent to defining the area of the peak (signal) at 25% of its height (Figure 5(a)). 

Raising the cutoff well above the baseline was done to provide a cleaner signal by excluding pixels on the periphery of the lesion with smaller fluctuations and to increase the likelihood that the region identified with a signal belonged to the lesion. Factors contributing to the noise can be technical and physiological in nature: the inherent limitations of the imaging technology (~15% scatter fraction reported for the 2D data acquisition mode) [29] as well as variations in the brain structure; in this particular case the uneven distribution of myelin within the different compartments of the brain, irregular delimitation of white matter, gray matter and lesions, and variation of myelin content inside the lesions.

Signal-to-noise ratio in imaging is generally defined as the ratio of the mean signal intensity and the standard deviation of the background intensity [30] (Figure 5):

(2) $$\text{SNR}=\frac{\mu_{\text{Signal}}}{\sigma_{\text{BGR}}}.$$

**Figure 6:**
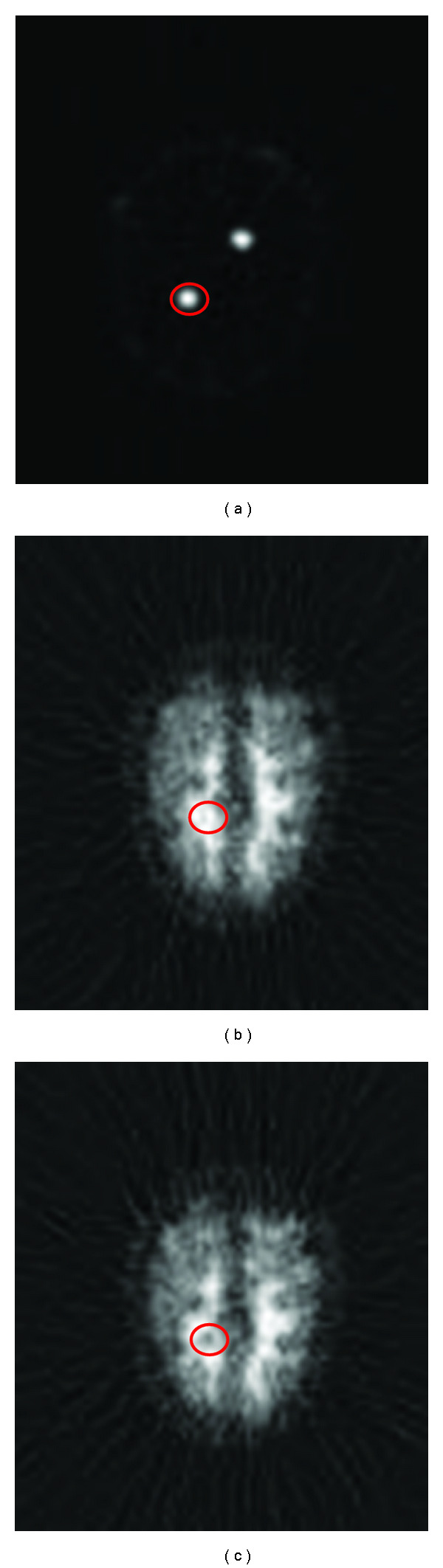
Identifying lesion location on simulated PET images with activity modeled only in lesions (a). The background intensity was calculated in the same area on the lesion-free image (b). The signal intensity was derived from the same area of the image containing the lesions (c). The area of interest is circled in red on (a, b, c). The lesion size in this series of images is 8 mm.

In the case of MS lesion imaging, the situation is different since the task is to detect a *decrease* in signal intensity in lesions inserted into a high activity background (“negative” signal, Figure 5(c)). As a consequence, we used the following modified formula for the signal-to-noise ratio calculation:

(3) $$\text{SNR}=\frac{\mu_{\text{BGR}}-\mu_{\text{LES}}}{\sigma_{\text{BGR}}},$$

where *μ*_BGR_ is the mean intensity of the background measured on the image without lesion, *μ*_LES_ is the mean intensity inside the lesion, and *σ*_BGR_ represents the fluctuations around the background (standard deviation). To evaluate the mean of the background signal, PET image simulations were run in pairs on the lesion-free and lesion-containing phantoms for every condition examined. After determining the lesion location and area on the lesion-only image (Figure 6(a)), as described above, the mean intensities were calculated for the corresponding pixels on the lesion-free (*μ*_BGR_) and lesion-containing images (*μ*_LES_) as shown in Figures 6(b) and 6(c). The standard deviation from the mean of the intensities on the lesion-free image (*μ*_BGR_) served as the measure of background noise (*σ*_BGR_). 

### 2.6. Biodistribution Scoring

In diagnostic imaging, a value of 3 to 5 for SNR is considered desirable in order to clearly delineate a region of interest [30]. For the purpose of this study, we considered a region with a SNR > 2 to be imageable. An SNR < 1 is considered to be not imageable since the signal is of comparable intensity to the noise. Lesions with SNR values between 1 and 2 might be imageable depending on a number of factors such as available reference (background), experience of observer, or software used for image analysis. These SNR cutoff values were set while assuming that coregistered MRI images were to be used to locate the lesions and define their areas. Once the location and accurate size of the region of interest (ROI) was determined, an algorithm would compare the coregistered PET signal originating from the ROI with the background signal of the normal-looking surrounding white matter. Instead of just relying on the ability of the human eye to detect the lesion, this would allow the use of simple statistical analysis to decide whether the region of interest has a significantly lower average signal relative to the background for a positive MS lesion identification.

## 3. Results

### 3.1. Acquisition Time

It is known that longer acquisition times result in sharper images [30], although they lead to increase in patient discomfort. To explore the effect of acquisition time on SNR, images of brain slices with 8 mm lesions simulated using 10 and 20 min acquisition times were compared. The result showed a 40% higher SNR for 20 min acquisition time (SNR = 1.01) relative to a 10 min one (SNR = 0.61, data not shown). Based on this finding, 20 min acquisition time was used for all subsequent simulations.

### 3.2. BioDMET Parameter Sensitivity Analysis

The PET image quality is primarily determined by the pharmacokinetics of the agent, which translates into activity ratios detected in white matter and gray matter relative to the lesions. The activity ratio, on its turn, is a direct reflection of the agent concentration ratios in these compartments. To better understand the agent and target properties that can affect imageability, a parameter sensitivity analysis was performed by varying the following input parameters: target concentration in the lesions, plasma protein binding, liver clearance, log *D*, specific binding affinity (*K*_*d*_), and binding on rate (*k*_*on* _) of the agent. Output parameters monitored were agent concentrations in the white and gray matter relative to the concentration in the lesions at 2 hours following administration, which corresponds to the image acquisition start time. The sensitivity coefficient of the output parameter *i* relative to the input parameter *j* was calculated according to the formula

(4) $$SC_{i,j}=\frac{\Delta Y_{i}}{\Delta X_{j}}$$

where Δ*Y*_*i*_ is the percent change in the output parameter *i* due to a Δ*X*_*j*_ relative change in input parameter *j*. Table 4 lists the input parameter values used in the calculations. 

**Figure 7:**
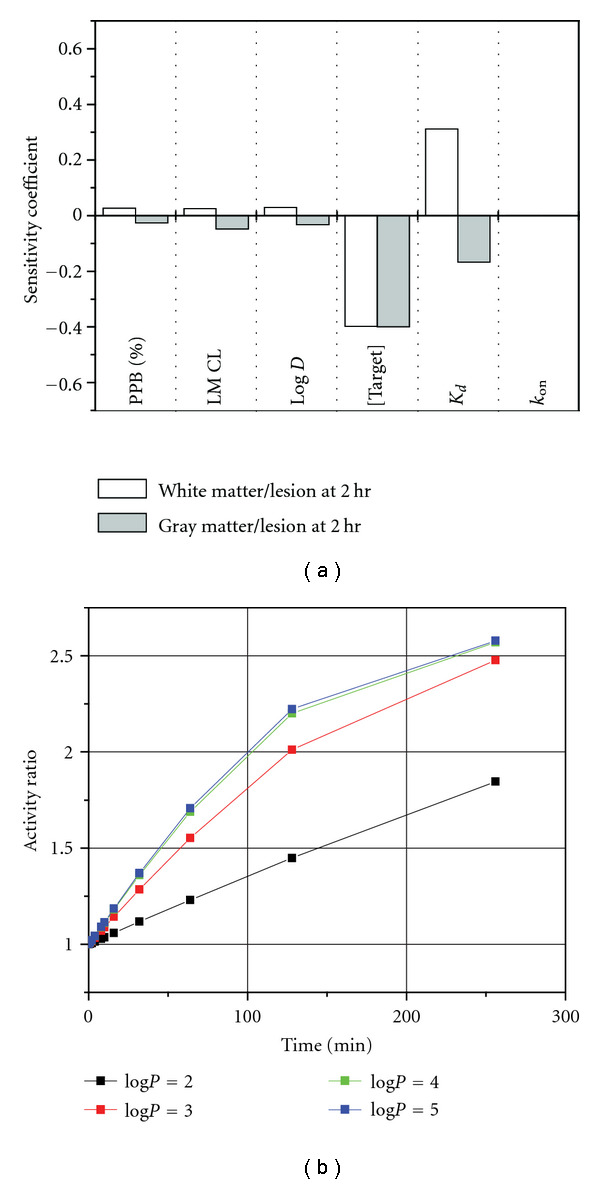
The effect of model input parameters on the activity differences in the white matter and the lesion. (a) Sensitivity coefficients relative to agent and target properties. (b) The influence of agent log *P* on the white matter-lesion activity ratio.

The agent concentration ratios in white matter versus lesion and gray matter versus lesion were found to be the most sensitive to the target concentration in the lesion and the agent binding affinity for the target as indicated by the values of the sensitivity coefficients (Figure 7(a)). 

The log *P* range of 4-5 examined in the sensitivity analysis corresponds to a highly lipophilic compound such as BMB, which is expected to partition significantly into the myelin of the brain. Decreasing the log *P* of the compound below a value of 4 would increase solubility but, at the same time, it is expected to negatively affect myelin partitioning. To verify this, the activity ratio in the white matter relative to the lesions was calculated for several values in the wider log *P* range of 2–5 as a function of time after administration. Not surprisingly, the effect of log *P* on the biodistribution ratio was found to be nonlinear and increasingly detrimental to the activity differences between these two regions. A drop of 2 log *P* units from 4 to 2 resulted in a roughly 40% decrease in the activity ratio between the white matter and the lesion. However, a one-unit decrease in the log *P* from 4 to 3 caused only a 10% decrease in the activity ratio (Figure 7(b)). For a myelin-specific agent, a log *P* above 3 seems to be necessary to produce significant differences between the white matter and MS lesions.

**Figure 8:**
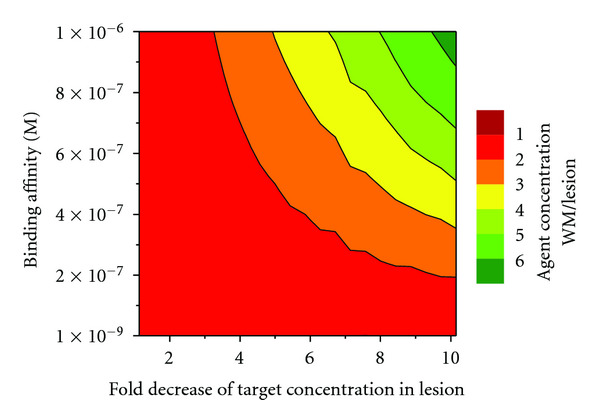
Heatmap of white matter/lesion agent concentration ratios as a function of target concentration in the lesion and agent binding affinity to the target. Imageability increases from red to green, with red regions corresponding to conditions that would make imaging impossible and green regions to conditions that would enable imaging of MS lesions imbedded in the white matter.

Next, we explored what regions of the parameter landscape could maximize the concentration ratios at 2 hours after agent administration, and, as a consequence, would maximize imageability. For this, 500 PBPK simulation runs were performed during which both the binding affinity and the target concentration in the lesions were sampled simultaneously using a Monte Carlo algorithm in the 10^−6^–10^−9^ M and 10^−4^–9 × 10^−4^ M range, respectively. The agent concentration ratio in the white matter relative to the lesions was monitored as the independent variable and the measure of imaging feasibility. As expected, more pronounced demyelination leading to lower target concentration in the lesions resulted in higher agent concentration ratios. Binding affinity showed a somewhat surprising trend: weaker, not stronger binding (higher *K*_*d*_) resulted in higher concentration ratios (Figure 8), suggesting better imageability.

### 3.3. Binding Affinity and Target Concentration Effects

**Figure 9:**
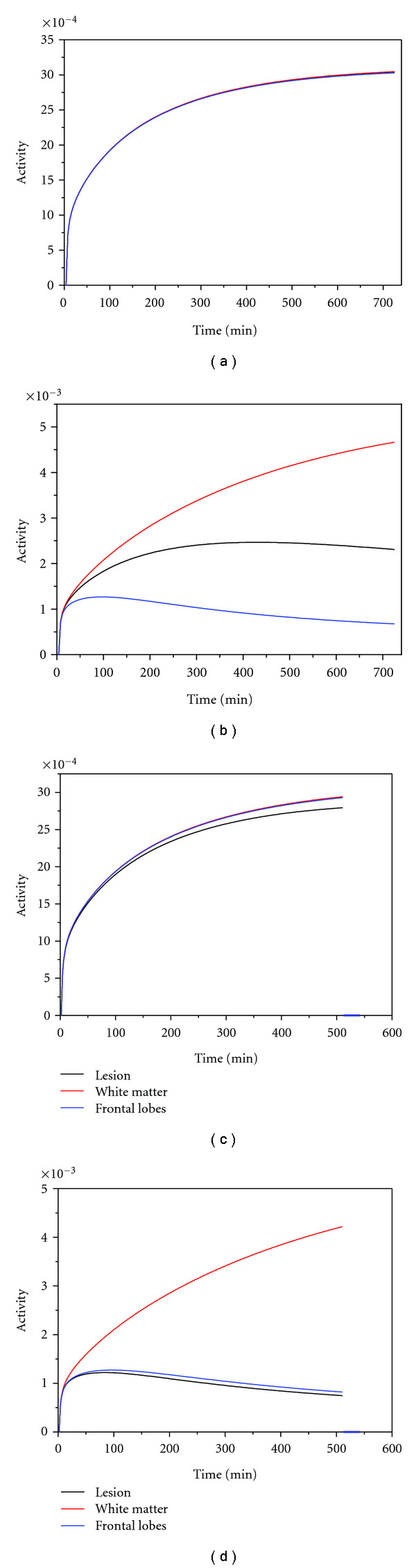
Time-activity curves for different *K*_*d*_ values and MBP concentrations in the lesions: (a) [MBP] = 3.61 × 10^−4^ M, *K*_*d*_ = 10^−9^ M; (b) [MBP] = 3.61 × 10^−4^ M, *K*_*d*_ = 10^−6^ M; (c) [MBP] = 1.015 × 10^−5^ M, *K*_*d*_ = 10^−9^ M; (d) [MBP] = 1.015 × 10^−4^ M, *K*_*d*_ = 10^−6^ M.

Agent concentration in white matter versus lesions is an important determinant of imageability, but it is not the only one. Lesion location, size, and inherent limitations of the methodology have to be taken into account as well. Therefore, several specific parameter combinations were used to calculate the corresponding agent biodistribution and generate simulated PET images for these conditions. The effect of lesion size and location was also evaluated. Lesions next to gray matter are expected to be more difficult to detect since the myelin content of the lesions is more similar to the myelin content of the gray matter than that of the white matter. Image analysis followed to determine the signal-to-noise ratios in the regions of interest containing the lesions inserted into the head phantom. Four sets of biodistribution (time-activity) curves are shown in Figure 9 as an example. The time-activity curves reflect the total activity resulting from both bound and free agent. Besides specific binding to the target, non-specific binding to proteins present in every tissue with a *K*_*d*_ = 10^−3^ M is also accounted for. 

Significant activity differences between the white matter and lesions could be observed only if the binding affinity was drastically decreased by three orders of magnitude (Figures 9(a) and 9(b)). At nanomolar binding affinities, the activity levels were found to be relatively insensitive to even a 35-fold additional decrease in MBP concentration in the lesions (Figure 9(c)). In contrast, an agent with micromolar binding affinity was predicted to be quite sensitive to a much smaller 3.5-fold additional decrease in MBP concentration within the lesions (Figure 9(d)). The activity levels remained unchanged upon 10-fold increases in *k*_*on* _ rates. 

**Figure 10:**
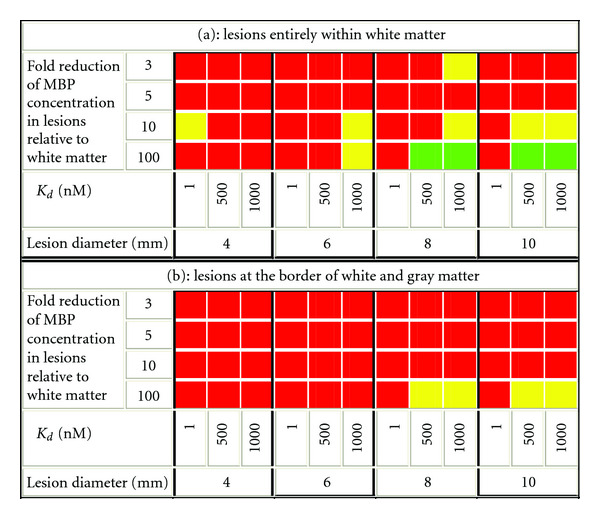
SNR values for the combinations of parameters explored. Red indicates SNR < 1 and is considered not feasible; yellow corresponds to 1≤ SNR < 2 and is classified as maybe feasible; green refers to cases with SNR ≥ 2 and is considered feasible.

Simulated PET images of the head phantom were generated with and without lesions, and SNR values were calculated for every lesion location in every parameter combination. The results shown in Figure 10(a) refer to lesions located entirely within the white matter, and in Figure 10(b) to the lesions near the border of white and gray matter. Imaging was considered to be not feasible for SNR < 1 (red), maybe feasible for 1≤ SNR < 2 (yellow), and feasible for SNR ≥ 2 (green). 

Based on the sets of conditions examined, imaging of MS lesions is predicted to be feasible under the following conditions:

- binding affinity of the imaging agent to the target in the micromolar range (0.5–1 *μ*M),
- myelin content of the lesions reduced at least 10-fold relative to the normal levels,
- lesions larger than 6 mm in diameter,
- lesions completely (or mostly) surrounded by white matter.

If the lower threshold of the SNR for “maybe” imageable is set to 1.3 instead of 1 (equivalent to a difference of 1.3*σ*_BGR_ between the mean of the signal and that of the noise), only lesions larger than 8 mm in diameter will be considered visible on PET images.

### 3.4. Accurate Lesion Localization

**Figure 11:**
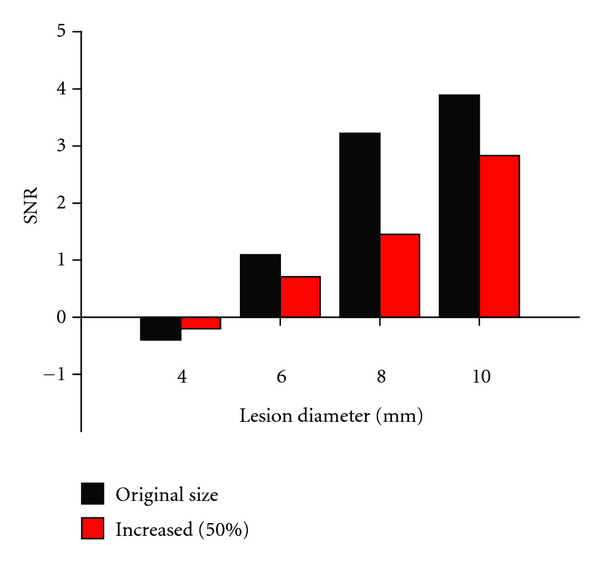
The effect of accurate lesions delineation on SNR. Relaxing the criteria of what pixels are included in the lesions to allow a 50% increase in lesion area resulted in 35–55% decrease in SNR.

To evaluate the effect of accurate determination of lesion size and location, the area of the lesions was increased 50% by including adjacent pixels in the determination of signal intensities both on the images with and without lesions. The calculations were done for the lesions completely embedded in the white matter with a 100-fold reduced myelin concentration and imaging agent binding affinity of 1000 nM. Signal-to-noise ratios decreased significantly for every lesion with 35–55% (Figure 11). 

## 4. Discussion

In clinical imaging applications, a key parameter is imageability, which is a complex function of biodistribution and clearance, and ultimately depends on the signal-to-noise (or signal-to-background) ratio achieved at the target at a particular point in time. In order to establish imageability criteria for reversible binders such as BMB, it is important to have a time frame sufficiently long for image acquisition during which a sustained concentration ratio between the target tissue and the background can be achieved. If this condition can be satisfied, as in our simulations, imageability criteria can be formulated in spite of the changing agent concentrations over time. Ultimately, as more experimental data is collected, the simulations can be further refined and the time-dependence of the signal-to-noise ratio can be determined more accurately.

Besides defining specific conditions under which imaging is predicted to be feasible, the present feasibility study provided a few additional insights detailed below.

### 4.1. Accurate Determination of Location and Size of the Lesions is Critical

MS lesions are usually sharply demarcated from the surrounding tissue in the MS brain when examined at autopsy. The limited resolution of PET imaging and the non-uniform distribution of the target in the brain, however, will result in decreased intensity spots with blurred borders on a background with uneven intensity. Since MS lesions are relatively small (median diameter 5-6 mm) and the change in the average signal intensity within the lesions is only about 10–30% when compared to the surrounding background, it will be a challenge to locate them on a PET image and determine their area accurately. Overcoming this challenge is important for a good signal-to-noise ratio, as shown earlier. Combining MRI and PET imaging might be the solution to this problem. The higher resolution of MRI would allow accurate localization of the lesions, whereas PET would enable specific measurement of their myelin content. Determining the background intensity could also be aided by MRI, which would be used to delineate the region corresponding to white matter. The average intensity of this region could serve as the reference to which the intensity of the lesions is compared. 

### 4.2. Stronger Binding Agent Might Not Always Be the Most Favorable

**Figure 12:**
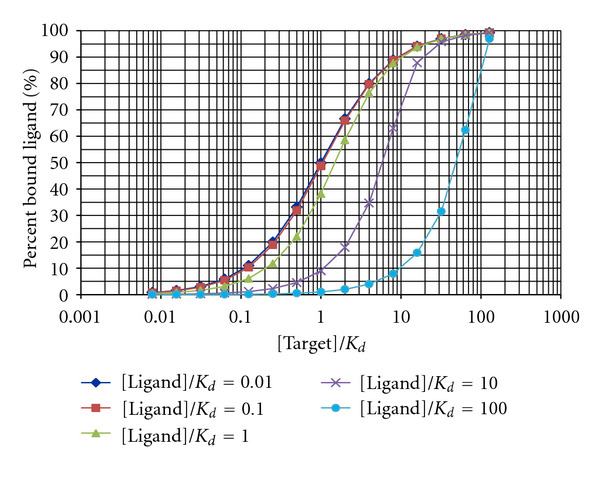
Protein-ligand binding kinetics: the fraction of bound ligand concentration depends on the total target and ligand concentrations and the binding affinity.

The fact that agents with lower *K*_*d*_ values (stronger binders) are worse than weaker binders when it comes to differentiating between regions of different target concentrations might not necessarily be intuitive. This somewhat unexpected behavior is due to the high target/agent concentration ratios both in the white matter and in the lesions and to the nonlinear, saturable kinetics of protein-ligand binding. The percent bound ligand as a function of the target and ligand concentration and the binding affinity is shown in Figure 12. Having a target concentration of approximately 10^−4^ M and a nanomolar binder ([Target]/*K*_*d*_ = 10^5^) with [Ligand]/*K*_*d*_ ratio of 1 (green curve in Figure 12) will result in 100% bound ligands in the lesions. The 10-fold excess of target concentration in the white matter will decrease the [Target]/*K*_*d*_  ratio to 10^4^, but this will still result in practically 100% agent binding. Since the target/agent concentration ratio is extremely high (~10^5^ to 10^6^), a 10-fold decrease in target concentration in lesions might decrease the bound ligand concentration with about 0.001%. The consequence is that the entire amount of the agent reaching the normal white matter, the gray matter or the lesions will bind, and no difference between the activity levels in these regions will be detected. Differential binding, and, consequently, different activities could be achieved if the target concentrations in the lesions were several orders of magnitude lower than in the normal appearing white matter or by using a considerably weaker binder as an imaging agent. Weaker binders, however, might result in loss of specificity. The optimal scenario would be to have an MS lesion-specific marker that is not present in the white matter. The binding affinities of ligands against such a marker are not subjects to upper constraints. These are the straightforward cases when the stronger the binding the better the SNR.

### 4.3. Longer Acquisition Times Might Be Necessary for Better Differentiation of Lesions

A compromise has to be reached between the patient discomfort caused by having to lie still inside the scanner for an extended period of time and the need for longer acquisition times to obtain sharper images of the lesions. Even a 20-minute acquisition time resulted in relatively weak SNR; decreasing this time to 10 minutes would weaken the signals about 40% to the point where imageability would not be feasible under any conditions. Increasing the acquisition time even further (to 30 minutes or higher) would most likely be too uncomfortable for the patients. Based on this study, a 20-minute acquisition time seems to be most reasonable. 

As a summary, our study indicates that PET imaging of MS lesions is far from being a trivial task. PET images would be useful by providing specificity for MS lesions, but they should be coregistered with MRI images to spot the lesions and delineate them accurately. Imaging agents that are relatively weak (micromolar) binders but specific for a component of myelin could be used to obtain discernable images of lesions with diameters of about 6 mm or larger embedded into the white matter if the myelin-specific target content of these lesions is at least 10-fold reduced relative to the surrounding tissue.

## Figures and Tables

**Table 1:** Target/agent properties modeled.

Variable	Range of values
Agent molecular weight	342 Da
Agent Log *P*	2–5
Agent administered amount	10^−9^ moles
Target	Myelin Basic Protein (MBP)
Target location	CNS myelin in white matter, gray matter and MS lesions
Target concentration	
White matter	1.015 × 10^−3^ M
Gray matter	1.345 × 10^−4^ M
MS lesions	1.015 × 10^−5^–3.61 × 10^−4^ M
Target molecular weight [25]	18.5 kDa
Agent-target binding affinity (*K*_*d*_)	10^−6^–10^−9^ M
Agent-target association rate (*k*_*on* _)	10^5^–10^6^ M^−1^s^−1^

**Table 2:** Tissue composition and target concentration in the modeled brain compartments.

	White matter	Gray matter	MS lesion
Mass fraction of brain (%)	42	56	2
Water fraction [21] (%)	72	82	76
Total protein (% dry weight) [21]	39	55.3	31.7
Total protein (% wet weight)	10.92	9.954	7.608
Total protein (g/g wet weight)	109.2 × 10^−3^	99.54 × 10^−3^	76.08 × 10^−3^
MBP (mg/g protein)	172 [24]	25 [23]	87.8 [24]
MBP (mg/g wet weight)	18.7824	2.4885	6.679824
MBP (Molar concentration)	1.015 × 10^−3^	1.345 × 10^−4^	3.61 × 10^−4^

**Table 3:** Sagittal (*x*), coronal (*y*), and transverse (*z*) coordinates of lesions inserted into the white matter of the Zubal head phantom.

Diameter (mm)	Location					
	Completely within the white matter			At the border or white and gray matter		
	*x*	*y*	*z*	*x*	*y*	*z*
4	103	123	52	142	144	52
6	106	84	63	144	86	63
8	110	105	45	139	140	45
10	151	127	58	109	141	58

**Table 4:** Input parameters used for the sensitivity coefficient calculations.

Run no.	Description	Plasma protein binding	Liver microsomal clearance rate	Log *P*	Target conc.	*K*_*d*_	*k*_ on_	Sensitivity coefficient	
								WM/Lesion	GM/Lesion
		(%)	(mL/min/mg protein)		(M)	(M)	(M^−1^s^−1^)		
(1)	Baseline	10	8 × 10^−3^	4	3.61 × 10^−4^	1 × 10^−6^	5.5 × 10^5^	—	—
(2)	% PPB	20	8 × 10^−3^	4	3.61 × 10^−4^	1 × 10^−6^	5.5 × 10^5^	2.693 × 10^−2^	−2.661 × 10^−2^
(3)	LM CL	10	8.8 × 10^−3^	4	3.61 × 10^−4^	1 × 10^−6^	5.5 × 10^5^	2.534 × 10^−2^	−4.785 × 10^−2^
(4)	Log *D*	10	8 × 10^−3^	5	3.61 × 10^−4^	1 × 10^−6^	5.5 × 10^5^	2.967 × 10^−2^	−3.242 × 10^−2^
(5)	Target conc.	10	8 × 10^−3^	4	5.42 × 10^−4^	1 × 10^−6^	5.5 × 10^5^	−3.984 × 10^−1^	−3.995 × 10^−1^
(6)	*K*_*d*_	10	8 × 10^−3^	4	3.61 × 10^−4^	2 × 10^−6^	5.5 × 10^5^	3.115 × 10^−1^	−1.668 × 10^−1^
(7)	*k*_*on* _	10	8 × 10^−3^	4	3.61 × 10^−4^	1 × 10^−6^	1.0 × 10^6^	−1.526 × 10^−5^	−6.893 × 10^−5^
